# Molecular Mechanisms of Colistin-Induced Nephrotoxicity

**DOI:** 10.3390/molecules24030653

**Published:** 2019-02-12

**Authors:** Zhibo Gai, Sophia L. Samodelov, Gerd A. Kullak-Ublick, Michele Visentin

**Affiliations:** 1 Department of Clinical Pharmacology and Toxicology, University Hospital Zurich, University of Zurich, 8006 Zurich, Switzerland; Zhibo.Gai@usz.ch (Z.G.); Sophia.Samodelov@usz.ch (S.L.S.); gerd.kullak@usz.ch (G.A.K.-U.); 2 Key Laboratory of Traditional Chinese Medicine for Classical Theory, Ministry of Education, Shandong University, Jinan 250355, China; 3 Mechanistic Safety, CMO & Patient Safety, Global Drug Development, Novartis Pharma, 4056 Basel, Switzerland

**Keywords:** acute kidney injury, colistin, mitochondria, nephrotoxicity, polymyxins, proximal tubule

## Abstract

The emergence of multidrug resistant (MDR) infections and the shortage of new therapeutic options have made colistin, a polymyxin antibiotic, the main option for the treatment of MDR Gram-negative bacterial infections in the last decade. However, the rapid onset of renal damage often prevents the achievement of optimal therapeutic doses and/or forces the physicians to interrupt the therapy, increasing the risk of drug resistance. The proper management of colistin-induced nephrotoxicity remains challenging, mostly because the investigation of the cellular and molecular pharmacology of this drug, off the market for decades, has been largely neglected. For years, the renal damage induced by colistin was considered a mere consequence of the detergent activity of this drug on the cell membrane of proximal tubule cells. Lately, it has been proposed that the intracellular accumulation is a precondition for colistin-mediated renal damage, and that mitochondria might be a primary site of damage. Antioxidant approaches (e.g., ascorbic acid) have shown promising results in protecting the kidney of rodents exposed to colistin, yet none of these strategies have yet reached the bedside. Here we provide a critical overview of the possible mechanisms that may contribute to colistin-induced renal damage and the potential protective strategies under investigation.

## 1. Introduction

**Figure 1:**
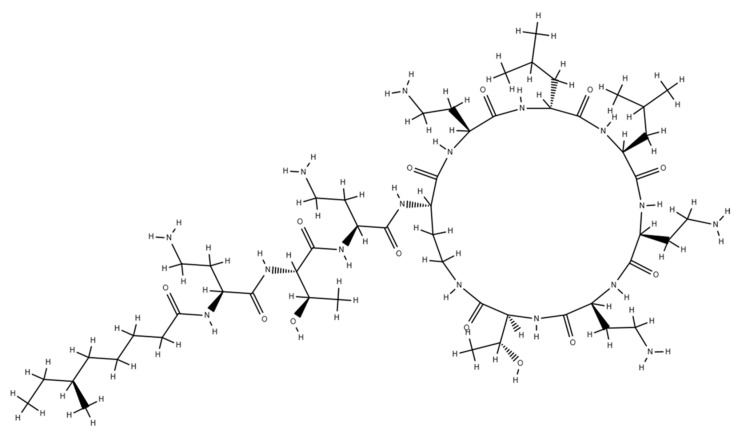
Chemical structure of colistin A (polymyixin E1). A cyclic heptapeptide with a tripeptide side chain acylated at the N-terminus by a fatty acid tail.

Colistin or polymyxin E was isolated in Japan from *Bacillus polymyxa* var. *colistinus* by Koyama [1]. From a chemical point of view, colistin shares a common backbone with polymyxin B: A cyclic heptapeptide possessing a tripeptide side chain acylated at the amino terminus by a fatty acid tail. Position 6 is occupied by D-phenylalanine in polymyxin B and by D-leucine in colistin (Figure 1). Colistin exerts its bactericidal effects mainly by disrupting the cell membrane integrity of the Gram-negative bacteria: through electrostatic interaction and cationic displacement (Ca^++^ and Mg^++^) of the lipopolysaccharide (LPS), colistin disturbs the stability of the membrane and increases its permeability, leading to the leakage of the cell content, triggering cell death pathways. Other bactericidal mechanisms of colistin may be (i) the neutralization of LPS, the endotoxin of Gram-negative bacteria and/or (ii) the inhibition of bacterial respiration [2,3].

In most of the reports published in its first decade of existence, colistin was demonstrated to have at least comparable potency to polymyxin B with less incidence of adverse reactions, especially nephrotoxicity, thus outranking polymyxin B and the other polymyxins with its wider therapeutic index. Colistin was introduced in the clinic in 1961 with the expectation, by the scientific community, of finally filling the void in the treatment of challenging infections such as those from antibiotic resistant *Pseudomonas aeruginosa* [4]. Shortly thereafter, side-by-side studies showed that colistin was not better tolerated than the other polymyxins, scaling down colistin use to levels comparable with polymyxin B [5,6]. With the discovery and approval of new, more tolerable antibiotics, colistin, like the other polymyxins, was gradually dismissed from clinical use due to the high incidence of nephrotoxicity.

Because of the increasing incidence of multidrug-resistant (MDR) bacterial infections, such as methicillin-resistant *Staphylococcus aureus*, vancomycin-resistant *enterococci* and some Gram-negative bacilli like *Pseudomonas aeruginosa*, *Acinetobacter baumannii* and *Enterobacteriaceae*, colistin has regained significant interest. Currently, colistin is considered the last-resort antibiotic in many areas where MDR is observed in medicine [7,8]. However, optimal administration of this antibiotic and the appropriate management of the related nephrotoxicity remain challenging, mostly because the pharmacology of this drug is still largely undescribed. We provide an overview of the clinical and histopathological characteristics of colistin-induced kidney damage and a critical review of the possible mechanisms underlying colistin-induced nephrotoxicity. Sources for this review were obtained through extensive literature searches of publications browsing PubMed. The main keywords used for searches were colistin, polymyxin E, nephrotoxicity, drug-induced kidney injury, and mitochondria. Only papers published in the English language were considered.

## 2. Drug-Induced Nephrotoxicity: An Overview

According to the clinical practice guidelines from Kidney Disease: Improving Global Outcomes (KDIGO), nephrotoxicity, in patients with normal renal function (serum creatinine of 1.3 mg/dL in women and 1.5 mg/dL in men), is apparent when one of the following are fulfilled: (i) increase in serum creatinine by ≥0.3 mg/dL within 48 h, (ii) increase in serum creatinine to ≥1.5 times baseline, which is known or presumed to have occurred within the prior 7 days, and/or (iii) urine volume <0.5 mL/kg/h for 6 h [9,10]. With the increasing use of medications over the years, drugs have become a substantial contributor to nephrotoxicity etiopathogenesis, including acute kidney injury (AKI) and chronic kidney disease (CKD). Prospective studies have established that drug-related nephrotoxicity accounts for 14–26% of all acute kidney injury cases in the adult population [11,12,13]. Analogously, 16% of the pediatric cases of AKI that required hospitalization have been attributed to the use of drugs [14]. Notably, there is no consensus on the definition of drug-induced kidney injury (DIKI), leading to challenges in the recognition and reporting thereof, especially for drugs not previously associated with kidney damage. Therefore, the incidence of DIKI may be underestimated. To harmonize the guidelines for the diagnosis of DIKI, a novel framework to approach drug-induced nephrotoxicity, focused on Risk assessment, early Recognition, targeted Response, timely Renal support and Rehabilitation coupled with Research (the 6R approach) has been proposed. For more details on this matter, we refer to the original work by Awdishu and Mehta [15].

One kidney contains over one million nephrons, the functional units of the kidney. Each nephron is composed of a glomerulus and a tubule. The glomerulus filters the blood retaining cells and large proteins, producing an ultrafiltrate mainly composed by small molecules, from nutrients to ions. The ultrafiltrate enters the tubule in which highly specialized cells at various segments (proximal tubule, Henle’s loop and distal tubule) contribute to modify the native urine by removing substances from the tubular fluid (reabsorption) or adding substances to the tubular fluid (secretion). Filtration, reabsorption, and secretion regulate the homeostasis of water, minerals, electrolytes, protons and nutrients, and clears the body of drugs, contrast agents and toxins [16].

**Figure 2:**
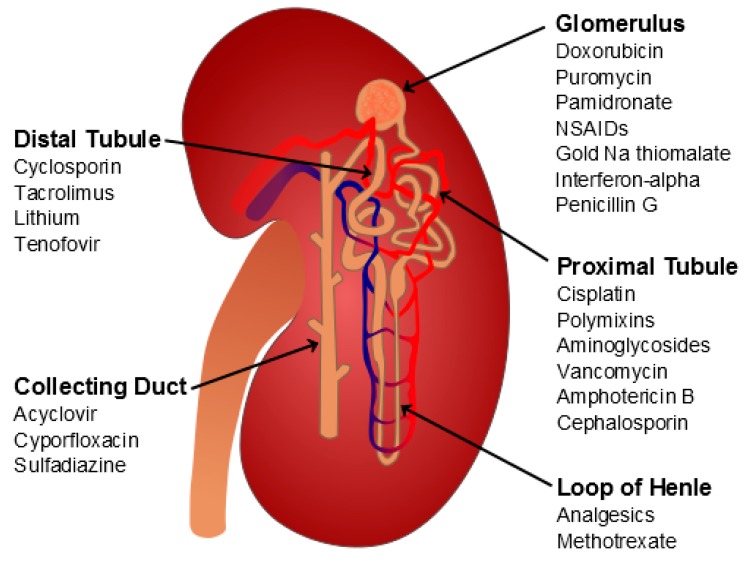
Main sites of drug-induced kidney injury. Schematic representation of a nephron, the functional unit of the kidney. Each nephron is composed of a glomerulus and a tubule. The glomerulus filters the blood, producing an ultrafiltrate mainly composed by small molecules, from nutrients to ions. The ultrafiltrate enters the tubule in which at various segments (proximal tubule, Henle’s loop and distal tubule) it is modified to obtain the final urine in the collecting duct.

The kidney damage induced by drugs can involve any of the nephron segments (Figure 2). Some drugs can cause a glomerular lesion (e.g., pamidronate, non-steroidal anti-inflammatory drugs, anti-angiogenetic drugs), others preferentially target the tubule. The injury can be cell-specific (e.g., proximal tubule cells) or can be the result of a nonselective insult to multiple cell types and damage of the epithelial cells throughout the tubule. The pathophysiology of drug-induced nephrotoxicity includes changes in glomerular hemodynamics, tubular cell toxicity, inflammation, crystal nephropathy (stone formation), rhabdomyolysis (breakdown of muscle fibers), and thrombotic microangiopathy (ischemic capillaries) [17,18,19]. An acute tubular damage of necrotic nature at the level of the proximal tubule cells is considered the primary event in colistin-induced kidney injury [20].

## 3. Clinical and Histopathological Manifestations of Colistin-Induced Nephrotoxicity

While nephrotoxicity was the main reason for colistin’s dismissal from clinical use in the first place, more recent clinical studies have diminished this issue by observing a lower incidence of kidney damage in patients treated with colistin as compared with initial studies. This discrepancy might be explained by the lack of common criteria to define renal function impairment and by the increased use of purer drug preparations [7,21]. The use of the KDIGO criteria to define AKI, rarely applied to patients receiving colistin, may help to better define the incidence of colistin-induced kidney damage. In a recent study, among 249 patients treated with intravenous colistin, rates of AKI using these clinical practice guideline criteria were 12% and 29% at 48 h and 7 days, respectively, from the initiation of the treatment. Seven percent of patients required renal replacement therapy following colistin initiation [22].

Another confounding factor is represented by the wide spectrum of dosing regimens used throughout the world, likely because the majority of dosing strategies currently in use represent the legacy of past empirical approaches, in the absence of accurate pharmacokinetic and pharmacodynamic data. Recent clinical studies demonstrated that most of the currently recommended colistin regimens are sub-optimal and that much higher doses should be administered to maximize the antibiotic activity and reduce the development of resistance [23]. In a recent study, after administering a loading dose, the authors utilized the maintenance dose algorithm to target a colistin steady-state concentration of 2.5 mg/L. From a pharmacodynamics perspective, this target concentration would be sub-optimal, with minimal bactericidal activity (based on the minimum inhibitory concentration calculated in vitro). However, the reported 44% rate of renal toxicity discouraged the authors from recommending higher target concentrations to increase the therapeutic response and to reduce the onset of colistin resistance [24].

Clinical manifestations of colistin nephrotoxicity include a decrease in creatinine clearance, as well as potential proteinuria, cylindruria (presence of casts in the urine), or oliguria (low output of urine) [25,26,27,28,29,30,31]. Nonetheless, assessment of histological abnormalities associated with colistin treatment, extensively reported in animal models, appear to be the most accurate way to diagnose colistin nephrotoxicity, albeit not doable within a clinical setting. Kidneys of rats treated with colistin for 7 days are marked by tubular dilation and epithelial cell vacuolation, tubular epithelial cell necrosis with numerous casts, but without evidence for an inflammatory response or fibrous cicatrisation [20]. There is little data regarding the best biomarker for the early detection of colistin nephrotoxicity. Use of the serum creatinine level for the estimation of the glomerular filtration rate (GFR) has some limitations, such as dependence on sex, age, nutrition and body mass, and is likely to reflect an already advanced damage. Studies in animal models showed that Cystatin C and kidney injury molecule 1 (KIM-1) might be more reliable markers than plasma creatinine to monitor renal function during colistin treatment [20,31]. Cystatin C is a cysteine protease inhibitor that is synthesized by all nucleated cells and freely filtered by the glomerulus, reabsorbed completely in proximal tubules, and not secreted under normal, healthy conditions [32]. KIM-1 is a phosphatidylserine receptor with an immunoglobulin-like domain that is expressed in normal proximal tubular epithelial cells and overexpressed upon acute tubular injury. Urinary KIM-1 has been reported to be specific to proximal tubular damage [33].

## 4. The Detergent Theory

**Figure 3:**
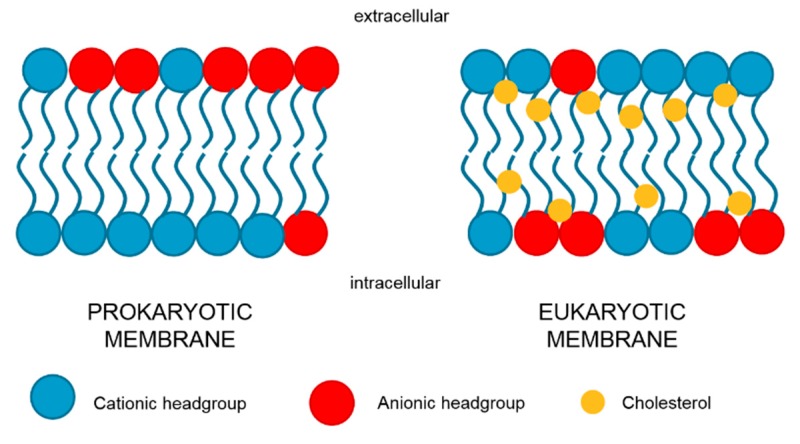
Lipid composition and distribution of prokaryotic and eukaryotic plasma membrane. Negatively charged phospholipids are enriched in the outer layer of prokaryotic membranes and in the inner layer of eukaryotic membranes. Cholesterol, a constituent of eukaryotic cells, stabilizes and protects the membrane from the colistin detergent activity.

Like the other members of the polymyxin family, colistin’s mechanism of action can be described by the “Shai-Matsuzaki-Huang (SMH) model”: antimicrobial peptides exert their bactericidal effects via interaction with the lipopolysaccharide (LPS) moiety of the membrane of Gram-negative bacteria (Figure 1). Electrostatic interaction with the negatively charged phospholipid headgroups and cationic displacement (Ca^++^ and Mg^++^) lead to disturbances in the stability of the membrane and an increase in its permeability, leakage of cell content, and cell death [34,35,36]. In 1970, Kunin found that polymyxin B and colistin sulfate tend to lose their antimicrobial activity when co-incubated with kidney tissue homogenate from rabbit. Sodium colistimethate, a less nephrotoxic species, was not sensitive to the incubation with the very same homogenate. The author concluded that polymyxin B and colistin sulfate, unlike colistimethate, are depleted by electrostatic interactions with the excess of anionic phospholipids contained in the homogenates. As for bacterial membranes, such interactions would destabilize the plasma membrane of eukaryotic cells with resulting leakage and death [37]. This “detergent theory” is mentioned in many reviews to explain colistin-induced nephrotoxicity, yet it is difficult to reconcile with the different biochemistry and physiology of prokaryotic and eukaryotic membranes (Figure 3). While both prokaryotic and eukaryotic membranes are enriched in anionic phospholipids, especially the renal brush-border membranes, their spatial organization within the lipid bilayer is substantially different. In bacterial membranes, the anionic phospholipids mainly localize at the outer leaflet with the negatively charged headgroup exposed to the extracellular milieu. In eukaryotic cells, negatively charged phospholipids are segregated into the inner leaflet of the membrane with the headgroup pointing towards the intracellular space. Hence, the interaction of the antibiotics with the plasma membrane of mammalian cells is limited in comparison with that of bacterial membranes [38]. Consistently, Mohamed and coworkers recently reported that colistin exposure induces haemolysis of red blood cells in culture but only at extracellular concentrations of 50 µM or higher, levels not achievable with the current colistin therapeutic regimens [39]. Furthermore, the effect observed by Kunin using tissue homogenates was not specific for the kidney. Similar results were obtained using liver or heart homogenates, tissues that are usually not sensitive to colistin in vivo. It is possible that the homogenization process renders the negatively charged phospholipids more accessible to colistin than in the intact cells [37]. Finally, it should be kept in mind that cholesterol, a component of the eukaryotic bilayer absent in bacterial membranes, tends to reduce the antimicrobial peptide activity by stabilizing the lipid bilayer or by directly interacting with the peptide [34,38]. Does the detergent theory still stand considering these fundamental differences in cell membrane composition? If so, a resulting prerequisite for colistin to be able to disrupt the plasma membrane integrity of eukaryotic cells would be an efficient uptake of the drug. Intracellular colistin would then have direct access to the acidic phospholipid components of the membrane and might indeed disrupt the membrane integrity.

## 5. Membrane Transport of Colistin

**Figure 4:**
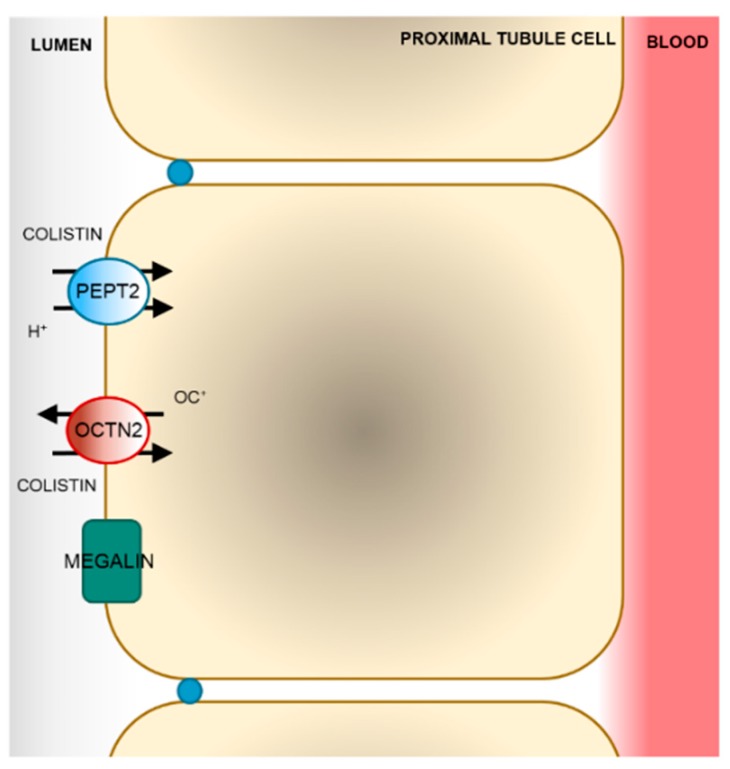
Model of colistin re-absorption in proximal tubule cells. After glomerular filtration, colistin is taken up by proximal tubule cells by facilitative transport mediated by the human peptide transporter 2 (PEPT2) and the carnitine/organic cation transporter 2 (OCTN2) and by the megalin-mediated endocytosis.

Tubular epithelial cells express a wide range of transporters, some of them representing an important route of cell entrance for several drugs (e.g., gentamicin and cisplatin). These transporters are highly expressed at the plasma membranes (either the basolateral or the brush border) of the tubular epithelium and sensitize the tubular cells to a number of drugs or other xenobiotics [40]. Colistin is extensively reabsorbed by the proximal tubule cells. Due to its polycationic nature at physiological pH values, colistin diffuses only poorly across the lipid bilayer. Thus, its tubular reabsorption is likely to involve one or more transport systems. Nevertheless, studies focused on the identification of the molecular mechanism of transport of colistin across the cell membrane are sparse and relatively recent. [41]. Currently, both endocytic processes and facilitative transport have been shown to contribute to the uptake of colistin across the apical side of proximal tubule cells (Figure 4).

In 2013, Suzuki and colleagues reported that colistin is reabsorbed by the proximal tubular cells upon binding to megalin, a 600 kDa glycoprotein highly expressed at the apical membrane of the proximal tubule cells. Wistar rats displayed a reduction in the renal accumulation and a simultaneous increased urinary excretion of colistin when co-administered with a megalin-shedding agent (maleic acid), megalin ligands (cytochrome c and FRALB), or an endocytosis inhibitor (colchicine). The lower accumulation of colistin in the kidney cortex was accompanied by a substantial decrease in urinary *N*-acetyl-β-D-glucosaminidase (NAG) excretion, a marker of renal tubular damage [42]. Megalin has been shown to mediate the reabsorption of other polycationic antibiotics such as aminoglycosides [43,44]. The work of Suzuki represents the first experimental evidence that colistin exerts its nephrotoxic effect upon accumulation in the cells. 

Recently, two solute carrier membrane proteins (SLCs) were shown to interact with colistin in vitro: The human peptide transporter 2 (PEPT2, *SLC15A2*) and the carnitine/organic cation transporter 2 (OCTN2, *SLC22A5*) [45,46]. A systematic screening in cells transiently transfected with the open reading frames (ORFs) of a number of facilitative carriers expressed at the luminal side of proximal tubule cells showed that the transport of the tritiated dipeptide glycyl-sarcosine ([^3^H]gly-sar) mediated by PEPT2 was inhibited by polymyxin B (IC_50_ = 18.3 ± 4.2 µM) and colistin (IC_50_ = 11.4 ± 3.1 µM). However, only the transport of polymyxin B was directly measured (K_m_ = 87.3 ± 11.3 µM) [45]. Because a transport inhibitor is not necessarily a substrate of the transporter as well [47], the transport of colistin mediated by PEPT2 should be directly assessed by analytical methods such as liquid chromatography-mass spectrometry (LC-MS) or using radiolabeled colistin. Shortly thereafter, HEK293 cells stably transfected with the ORF of the OCTN2 were shown to accumulate more colistin over time than the respective wild-type cells [46]. OCTN2 is highly expressed at the luminal membrane of proximal tubule cells and is the major carrier of carnitine. The transport of carnitine mediated by OCTN2 is Na^+^-dependent, whereas that of other organic cations (e.g., tetraetylammine, TEA) is independent on Na^+^ [48]. The replacement of NaCl with choline chloride in the transport buffer did not alter the accumulation of colistin in the cells overexpressing OCTN2, indicating a Na^+^-independent transport of colistin [46].

A number of lines of evidence indicate that OCTN2 plays a central role in cellular carnitine uptake. Indeed, loss of function mutation in the *SLC22A5* gene causes systemic carnitine deficiency (OMIM 212140) [49]. A recent characterization of the urinary metabolites of colistin-induced nephrotoxicity in rats showed that a single intraperitoneal administration of colistin did not result in signs of renal damage but a 2-4-fold increase in the levels of a number of amino acids (e.g., isoleucine, valine) and carnitine derivatives in urine (e.g., acetylcarnitine, butyrylcarnitine) [50]. This supports the speculation for a role of PEPT2 and OCTN2 in colistin reabsorption in vivo. Nevertheless, to better characterize the actual contribution of PEPT2 and OCTN2 in colistin renal reabsorption, knock-out animals like the kidney-specific Pept2-null mice or the *jvs* mouse strain, which carries a spontaneous missense mutation in the *Slc22a5* gene, should be used in further studies [51,52].

## 6. The Intracellular Fate of Colistin

The information gathered in the last five years on colistin uptake pathways suggests that colistin exerts its toxic effect upon entering the cells. The high expression level of megalin, PEPT2, and OCTN2 at the luminal side of proximal tubule cells would imply that the accumulation of colistin is particularly high in these cells, providing an explanation for kidney sensitivity to colistin [53,54,55,56]. Using rationally designed fluorescent probes, Yun and coworkers confirmed that polymyxins accumulated in the proximal tubule cells [57]. Mice treated with 7.5 or 15 mg of colistin/kg of body weight/day for 7 days showed signs of apoptosis involving mitochondrial Bcl-2 and Bax, death receptor (upregulation of Fas, FasL, and Fas-associated death domain), and endoplasmic reticulum (ER) pathways (upregulation of Grp78/Bip, ATF6, GADD153/CHOP and caspase-12), suggesting that mitochondrial and ER stress upon colistin exposure [58].

**Figure 5:**
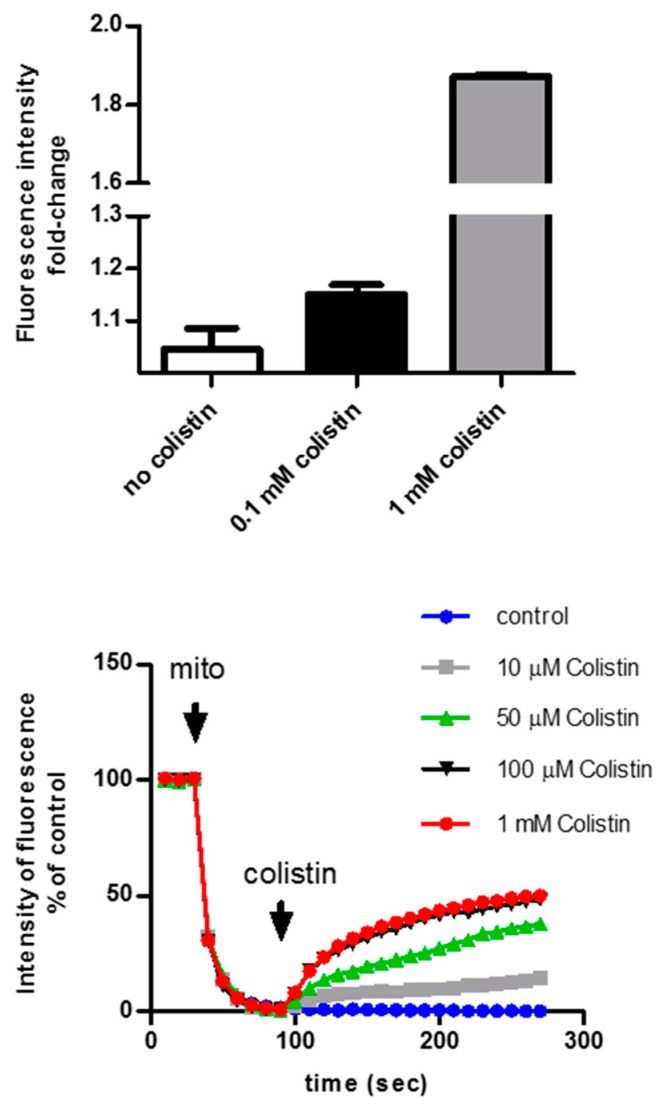
Effect of colistin on isolated mitochondria. Freshly isolated mitochondria from mouse kidneys were exposed to a hydrophilic Ca^2+^-sensitive dye: calcium release from the mitochondria results in an increase in the fluorescent signal (top panel). Mitochondria were exposed to rhodamine 123, a membrane potential-sensitive) dye. The lower the membrane potential, the higher the rhodamine 123 fluorescence signal (λ_ex_ = 488 nm, λ_em_ = 527 nm) (bottom panel).

Studies addressing the nature of colistin-induced mitochondrial dysfunction have not yet been completed. Indeed, neither the in vitro nor the animal studies available can distinguish between primary and secondary mitochondrial and ER dysfunction induced by colistin. To address this question, experiments in freshly isolated mitochondria should be performed. In our lab, we isolated intact mitochondria from mouse kidneys and exposed them to increasing concentrations of colistin. The mitochondria rapidly depolarized in a dose-dependent manner, suggesting that colistin directly targets the mitochondria (unpublished data) (Figure 5).

The target of colistin in mitochondria is not known but interesting hints may be gathered from the studies focused on elucidating the mechanisms of colistin’s antimicrobial activity. Polymyxins inhibit cellular respiration in Gram-negative and Gram-positive bacteria [59,60]. The prokaryotic respiratory chain consists of three complexes with quinones and reduced nicotinamide adenine dinucleotide (NADH) shuttling electrons and protons between large protein complexes [61,62]. In complex 1, three inner membrane respiratory enzymes of the NADH oxidase family have been identified: proton-translocating NADH-quinone (Q) oxidoreductase (NDH-1), NADH-Q oxidoreductase (NDH-2), which lacks an energy-coupling site, and the sodium-translocating NADH-Q oxidoreductase [63]. Deris and colleagues reported that colistin and polymyxin B inhibit the NDH-2 activity in a non-competitive manner [3]. NDH-2s are the only enzymes performing respiratory NADH:quinone oxidoreductase activity. For this reason and for being considered absent in mammals, NDH-2s were proposed as suitable targets for novel antimicrobial therapies [64]. However, a recent phylogenetic analysis clustered the human protein apoptosis-inducing factor-homologous mitochondrion associated inducer of death, AMID (AIF-M2) with the prokaryotic NDH-2 family and not in the group containing the canonic AIF proteins. AIF-M2 has been shown, indeed, to have NADH-Q oxidoreductase activity [65,66,67]. Overall, the effect of colistin on the human AIF-M2 protein and, in general, on the mitochondrial electron transport chain should be further studied.

## 7. Protective Strategies

Although many risk factors for colistin-induced kidney injury have been identified, including advanced age, concomitant administration of nephrotoxins (e.g., aminoglycosides), and obesity, data supporting protective strategies that can widen the therapeutic window of this antibiotic are mostly anecdotal [68,69]. The growing evidence that colistin exposure induces oxidative stress in proximal tubule cells suggests that a concomitant anti-oxidant strategy could protect the kidney during the colistin exposure [58]. In the last decades, several compounds have been tested in animal models as protective agents against colistin nephrotoxicity (Table 1). While most of them were able to protect the kidney during colistin exposure, only ascorbate was tested in a small, preliminary, randomized clinical trial. The protective effect exerted by ascorbic acid against colistin-induced kidney damage in animals indirectly supports the hypothesis that colistin can impair the electron flux along the mitochondrial inner membrane. Ascorbic acid can replace NADH as electron donor [70].

Ascorbic acid is a potent reducing agent and radical scavenger and reduces stable oxygen, nitrogen, and thyl radicals [71]. It is believed to sustain the electron transfer across the mitochondrial inner membrane downhill of the complex IV [72]. 28 patients received colistimethate sodium (CMS) intravenously at a loading dose of 300 mg (∼10 million IU). Ascorbic acid was administered intravenously at a dose of 2 g every 12 h, 20 min before CMS, to 13 out of 28 patients (colistin-ascorbic acid group). Nephrotoxicity was defined by the Risk, Injury, Failure, Loss of kidney function, and End-stage kidney disease (RIFLE) classification system. Urinary neutrophil gelatinase-associated lipocalin (NGAL) and N-acetyl-β-D-glucosaminidase (NAG) were also assessed as markers of renal damage. The incidence of acute kidney injury was 53.8% and 60.0% (*P* = NS) in the colistin-ascorbic acid and colistin groups, respectively. Similarly, the excretion rates of NGAL and NAG, assessed at different time points, were not significantly different between the colistin-ascorbic acid group and the colistin group [73]. The study failed to demonstrate the protective role of ascorbic acid in colistin-induced kidney toxicity [73]. While the clinical study tried to mirror the regimen used in the animal study, it is not possible to control the intracellular level of colistin and ascorbic acid. It should be kept in mind that the intracellular accumulation of colistin and ascorbate might differ between species. Different ascorbate regimens might amplify the small, not significant, protective effect observed in this study.

## 8. Conclusions

With the currently available drugs, rate of development and approval of new antibiotics, and the increasing incidence of MDR infections, we will continue to rely on colistin as a last resort treatment in severe cases of Gram-negative infection within the near future. Patients under colistin treatment often suffer from other co-morbidities, not to mention having histories of longer-term hospitalization. It is of particular importance to focus our studies on the molecular and biochemical mechanisms of colistin-induced nephrotoxicity in order to facilitate the achievement of the best-possible colistin therapy outcome: loading dose to reduce or even clear the infection in the shortest possible time, limiting resistance development. Due to the relatively high incidence and quick onset of nephrotoxicity by treatment with colistin, it is important to (i) more fully understand the molecular effect of colistin on eukaryotic cells, (ii) elucidate its cellular transport and sub-cellular accumulation, (iii) establish founded dosing regimens in humans, based on rigorous PK/PD studies rather than empirical evidence, (iv) and either uncover novel protective agents or establish optimized dosing and application strategies for potential candidates. This review has discussed some essential old and new findings in colistin research that can be of particular interest in achieving these goals. Colistin’s effect on lipid membranes, particularly effects on mitochondria in eukaryotic cells, as well as the potential transporters that may play a role in colistin accumulation in the kidney cortex should continue to be studied further. Investing in such research projects for this relatively old drug is rightly justified due to the current necessity of colistin therapy in a clinical setting for severe MDR infections. Information resulting from such targeted studies would be of great help for clinicians to put the most efficient curative and protective strategies for colistin treatment into place.

## Figures and Tables

**Table 1:** Effect of antioxidant strategies on colistin pharmacokinetics (PK) and kidney function.

Compounds	Species	Colistin PK	Kidney Function	Reference
Ascorbate	rat	⇑ AUC ⇑ vd	⇓ NGAL ⇓ apoptosis	[74]
	human	⇔ AUC	⇔ NGAL ⇔ NAG	[73]
α-Tocopherol	rabbit	ND	⇓ sCRE ⇓ BUN	[75]
Baicalein	mouse	ND	⇓ BUN ⇓ sCRE	[76]
Cilastatin	mouse	ND	⇓ NGAL ⇓ KIM-1	[77]
Curcumin	rat	ND	⇓ MDA ⇓ NO ⇓ inflammation	[78]
Gelofusin	mouse	ND	⇓ necrosis ⇓ inflammation	[79]
	rat	⇔ AUC ⇔ vd	ND	
Proanthocyanidin	rat	ND	⇓ BUN ⇓ sCRE ⇓ iNOS	[80]
Luteolin	rat	ND	⇓ sCRE ⇓ apoptosis	[81]
Lycopene	mouse	ND	⇓ sCRE ⇓ BUN ⇓ apoptosis ⇓ necrosis	[82]
Melatonin	rat	⇑ AUC ⇑ vd	⇓ NGAL ⇓ sCRE	[83]
*N*-acetylcysteine	rat	ND	⇓ SOD ⇓ NO	[84,85]
Sylibin	rat	ND	⇓ NAG ⇓ Urea ⇓ sCRE ⇓ Urate ⇓Na^+^, K^+^	[86]
Taurin	mouse	ND	⇓ sCRE ⇓ BUN	[87]
Vitamin E	rat	ND	⇓ uGGT ⇓ MDA	[88]

AUC, area under the curve; BUN, blood urea nitrogen; MDA, malondialdehyde NAG, *N*-acetyl-β-D-glucosaminidase; ND, not determined; NGAL, neutrophil gelatinase-associated lipocalin; NO, nitric oxide; sCRE, serum creatinine; SOD, superoxide dismutase; uGGT, urine γ-glutamyl-transferase; vd, volume distribution.
